# Steady state trials: another valid substitution of counterfactual ideal to measure causal effects

**DOI:** 10.1007/s12199-012-0312-8

**Published:** 2012-10-31

**Authors:** Okujou Iwami, Masayuki Ikeda

**Affiliations:** 1 Iwami Neurological Clinic, 1-5-2 Ohdohri, Miyako, 027-0083 Japan; 2 Kyoto Industrial Health Association, 67 Nishinokyo-Kitatsuboicho, Nakagyo-ku, Kyoto, 604-8492 Japan

**Keywords:** Cross-over trials, Counterfactual model, Steady state, Period ratio, Individual causal effect

## Abstract

**Objectives:**

Many traditionally established medical interventions are not examined with randomized trials especially in emergency medicine. We researched what is the scientific basis of the measurement of the causal effect in these interventions and proposed another trial to measure causal effects.

**Methods:**

We deduced steady state trials from the counterfactual model and used Bayesian approaches to estimate causal effects statistically.

**Results:**

When the state of the observed person is fairly steady before an exposure, the ratio of the after-period to the before-period of the exposure is sufficiently small, and changes are obtained in relatively short time, it is possible to postulate that the state of the counterfactual person to be compared is almost equal to the state of the real person before the exposure. Bayesian approaches show that the causal effect of the exposure is estimated even in only one-person steady state trials, when large changes are observed.

**Conclusions:**

Steady state trials are valid methods to measure causal effects and can measure causal effects even in one-person trials. When we can measure the causal effect of interventions with steady state trials, these interventions should be regarded as scientific without use of randomized trials.

## Introduction

Evidence-based medicine (EBM) appeared as a handy tool kit for clinicians who had not understood the basic thinking of epidemiology [1]. After the advocates of EBM succeeded in nominating randomized trials to be paramount [2], the so-called “Hierarchy of Strength of Evidence” towered in medical practice and many clinical guidelines prostrated themselves in front of the pyramid [3, 4]. Many traditionally established medical interventions were stripped of their rank for reasons having to do with observational studies. Under these circumstances, Smith and Pell [5] asked a sarcastic question why protagonists of EBM did not participate in a randomized trial of parachute use.

In epidemiological studies, the counterfactual or potential-outcome model has become increasingly standard for causal inference [6–8]. However, the theoretical ideal to measure causal effects of exposure is impossible. To achieve a valid substitution for the counterfactual experience, we resort to various design methods that promote comparability. One approach is a cross-over study and another is a randomized trial. Other approaches might involve choosing unexposed study subjects who have the same or similar risk-factor profiles for disease as the exposed subjects [9]. Case-crossover design was introduced for estimating a short term, transient effect of intermittent exposures on acute-onset diseases [10, 11]. For each case, one or more predisease or postdisease time periods are selected as matched control periods for the case. The exposure status of the case at the time of the disease onset is compared with the distribution of exposure status for the same person in the control periods. The key feature of the case-crossover design is that each case serves as its own control. In this paper, we expand this key feature and propose another valid substitution of the counterfactual ideal to measure causal effects and show that parachute use and many interventions in emergency medicine have the scientific basis of the causal inference without randomized trials.

## Materials and methods

**Fig. 1:**
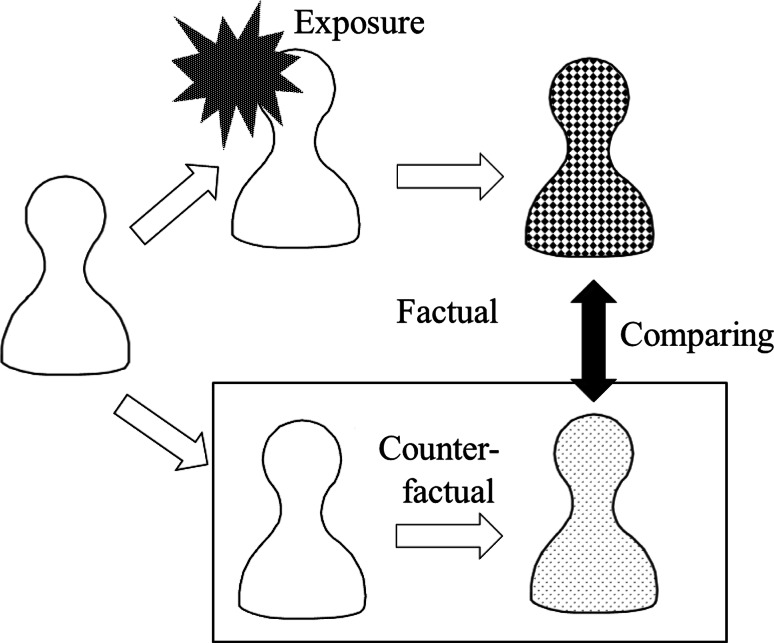
Counterfactual model. We establish a hypothetical person in the counterfactual world in order to compare the outcome of the exposed person with the outcome of the unexposed person. After the exposure, both the conditions of the exposed person and the unexposed person are observed at the same time. As the only difference between the two settings is the exposure, it is possible to measure the effect of the exposure

We deduce steady state trials from the counterfactual model. The scheme is presented in Fig. 1. Bayesian methods are used to estimate causal effects statistically [12, 13] (see appendix). Posterior distributions are computed with WinBUGS version 1.4.3, which reports two-sided equi-tail-area credible intervals [14]. We use these intervals for convenience, although highest posterior density intervals are more preferable.

## Results

### Steady state trials

For the purpose of discussion, letters are defined as follows;

*t*: time
*T*_0_: the time when the observation starts
*T*_1_: the time when the exposure is done
*T*_2_: the time when the outcome is observed
*B* = (*T*_1_ − *T*_0_): the period before the exposure
*A* = (*T*_2_ − *T*_1_): the period after the exposure
*n*: the integer which gives the ratio of *A* to *B*, *A*:*B* = 1:*n*
*S*: the state of the observed person which is a function of time
*X*: the state *S* just before the time *T*_1_
*Y*: the state *S* at the time *T*_2_
*Z*: the state of the counterfactual ideal of the unexposed person which is a function of time
*W*: the state *Z* at the time *T*_2._

**Fig. 2:**
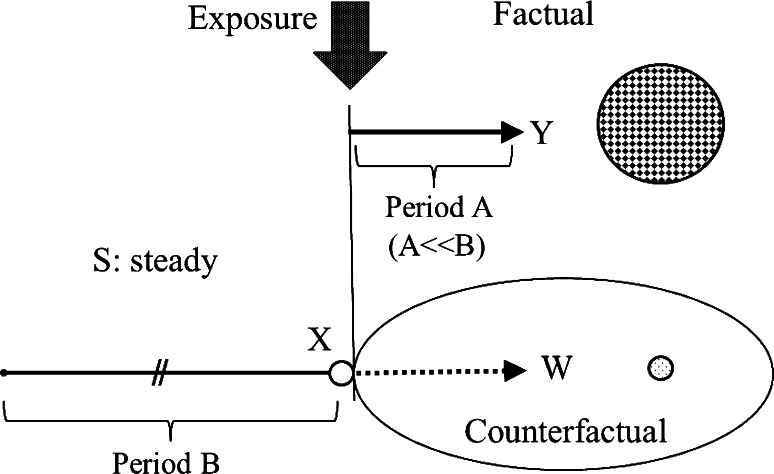
Steady state trials. *S* the state, *X* *S* just before the exposure, *Y* *S* at the end of the period *A*, *W* the counterfactual state at the end of the period *A*. *S* is steady during the period *B*

**Fig. 3:**
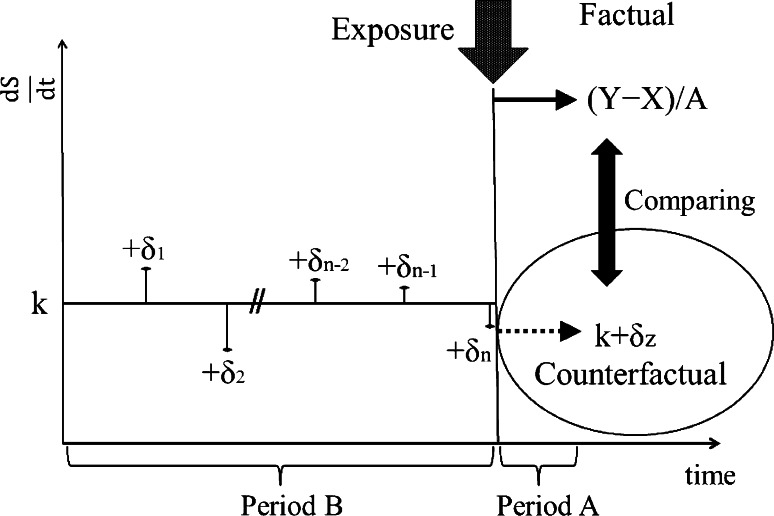
Derivative of the state. *Period A*:*Period B* = 1:*n*, where *n* is a positive integer. $\frac{{{\text{d}}S}}{{{\text{d}}t}}$ is the derivative of *S* with respect to time; $\frac{{{\text{d}}S}}{{{\text{d}}t}}$ = *k* + δ, where *k* is constant and δ (δ_*i*_; *i* = 1, 2,…, *n*) is noise which follows the normal distribution. We observe the state (*n* + 1)-times at the interval of the period *A* during the period *B*. The counterfactual derivative of the state is postulated as (*k* + δ*z*), where δ*z* follows the same distribution as δ_*is*_

Steady state trials begin with the observation of the state of the object person (Fig. 2). Suppose the state is almost steady during the period *B* (Fig. 3). Namely, the derivative of the state with respect to time during the period *B* is

$$ \frac{{{\text{d}}S}}{{{\text{d}}t}}  = k + \delta , $$

where *k* is a constant and δ is noise which follows the normal distribution *N*(0, σ^2^). We observe the state *S* (*n* + 1)-times at the interval of the period *A* and obtain sample noises *n*-times (δ_*i*_; *i* = 1, 2, …, *n*) during the period *B*. Just before the exposure, the state is recorded as *X*. When we observe *Y* at the end of the period *A* after the exposure, we get the mean value of $\frac{{{\text{d}}S}}{{{\text{d}}t}}$ during the period *A*:

$$ \frac{{{\text{d}}S}}{{{\text{d}}t}}  = (Y - X)/A. $$

When the ratio of the period *A* to the period *B* is sufficiently small, i.e., *n* is sufficiently large, we can postulate the derivative of the counterfactual unexposed state is *k* plus noise δ*z* which follows the same distribution as δ_*is*_:

$$ \frac{{{\text{d}}Z}}{{{\text{d}}t}}  = k + \delta z. $$

However, we cannot really observe $\frac{{{\text{d}}Z}}{{{\text{d}}t}}$, so the value of (*k* + δ*z*) is replaced with the observed value of (*k* + δ_*is*_). In order to estimate the difference between (*Y* –*X*)/*A* and (*k* + δ_*is*_), we postulate that the distribution of (*Y* – *X*)/*A* follows the normal distribution with the same variance as σ^2^ which is estimated by the sample variance of (*k* + δ_*is*_). Then the difference between (*Y *–* X*)/*A* and (*k* + δ_*is*_) can be statistically estimated with the *t* distribution. When the outcome *Y* has the quality different from the state *X*, the nominal scale is applied.

### Statistical inference of causal effects

Suppose when we observe the outcome *Y* which belongs to the category different from the state *X* and the change is of practical importance, or when the difference between (*Y *– *X*)/*A* and (*k* + δ_*is*_) is statistically significant and large enough to be of practical importance. We now discuss the causation of the incidence of such an important outcome *Y*, which we designate *Y*_imp_ in the following discussion.

The probability that *Y*_imp_ happens during the period *A* with the exposure is represented by the letter *θe*:

$$ \theta e = \, P(Y_{\text{imp}} \, | \, E, \, C), $$

where *E* is the exposure, *C* is the condition that the state is steady during the period *B* and the vertical line represents conditioning. We postulate that *Y*_imp_ is a Bernoulli variable during the period *A*. The probability that *Y*_imp_ happens during the period *A* without the exposure (¬*E*) is represented by the letter *θu*:

$$ \theta u = \, P(Y_{\text{imp}} \, | \, \neg E, \, C). $$

As the state is steady and the period *A* is small relative to the period *B*, we can postulate that the counterfactual condition of the unexposed state in the period *A* is equivalent to the real condition in the period *B*, and the probability that *Y*_imp_ happens within the time span of the period *A* during the period *B* is equal to *θu*. Then the period *B* has a sequence of *n*-times repetitions of a trial with constant probability *θu*.

Suppose that we observe *Y*_imp_ after the exposure and there is no incidence of *Y*_imp_ during the period *B* in one steady state trial. The components of the Bayesian model for steady state trials can be written as follows:

$$ \begin{gathered} {\text{prior distribution}}\,\,\,\,\,\theta e\,\,\,\,\,\,\,\sim {\text{ Beta}}\left( {\alpha_{ 1} , \, \beta_{ 1} } \right) \hfill \\ \,\,\,\,\,\,\,\,\,\,\,\,\,\,\,\,\,\,\,\,\,\,\,\,\,\,\,\,\,\,\,\,\,\,\,\,\,\,\,\,\,\;\;\;\;\theta u\,\,\,\,\,\,\,\sim {\text{ Beta}}\left( {\alpha_{ 2} , \, \beta_{ 2} } \right) \hfill \\ {\text{likelihood}}\,\,\,\,\,\,\,\,\,\,\,\,\,\;\;\;\;\;p\left( {{ye}|\theta e} \right) = \theta e \hfill \\ \,\,\,\,\,\,\,\,\,\,\,\,\,\,\,\,\,\,\,\,\,\,\,\,\,\,\,\,\,\,\,\,\,\;\;\;\;\;\;\;\;p\left( {{yu}|\theta u} \right) \, = \left( { 1- \theta u} \right)^{\text{n}} \hfill \\ {\text{posterior distribution}}\,\, \, \Updelta = \theta e\left| {{ye} - \theta u} \right|yu, \hfill \\ \end{gathered} $$

,where* ye* is the success of *Y*_imp_ in one trial under the exposure,* yu* is the no success of *Y*_imp_ in *n* trials under the non-exposure, and Δ is the difference between *θe* and *θu* taking account of the trial evidence. Ideally, the likelihood of no success under the non-exposure should be computed with *n*-times trials in the real world and one-time trial in the counterfactual world. The trial in the counterfactual world cannot be observed. Thus, we approximately compute this likelihood with *n*-times trials in the real world. The posterior distribution Δ is computed with WinBUGS.

When we are uncertain about the prior distribution, we adopt Beta(0.5, 0.5) or Beta(1, 1) as the reference prior distribution for *θe* and *θu*. The posterior distribution of *θu* shifts to zero as *n* becomes larger. With the reference prior Beta(0.5, 0.5) for *θe* and *θu*, the lower limit of the 95 % credible interval of Δ is over zero when *n* is equal to or more than four. With the reference prior Beta(1, 1) for *θe* and *θu*, the lower limit of the 95 % credible interval of Δ is over zero when *n* is equal to or more than seven. Classical statistical approaches also show similar results [15, 16]. The larger the number of *n* is, the more credibility we can gain in the inference of the causal effect. However, the lower limit of the 95 % credible interval of Δ cannot be over 0.15 with the prior Beta(0.5, 0.5) and not over 0.16 with the prior Beta(1, 1), no matter how *n* may become large. This is the limitation of one-person trials. Population studies with many persons can show larger lower limits of the credible interval, if the success proportion is high.

Steady state implies that the previous observations of the same condition showed no incidence of *Y*_imp_ without the exposure. When we believe the previous evidence for the no-incidence of *Y*_imp_ under the non-exposure, we can adopt, for example, Beta(1, 1000000) as the prior distribution for *θu*. Adopting almost null prior distribution Beta(1, 1000000) for *θu* means that *p*(Δ) is practically equal to *p*(*θe*|*ye*).

### Relation to cross-over trials

The simple cross-over design is outlined by Armitage et al. [17]. With two treatments, F and G, one randomly chosen group of patients receives treatments in the order FG, while the other group receives them in the order GF. The active response that is common to all subjects in a particular group and particular period with the treatment received is modeled as follows:

**Table:** 

	Period 1	Period 2
Group I (FG)	μ + τ_F_ + π_1_	μ + τ_G_ + π_2_ + γ_FG_
Group II (GF)	μ + τ_G_ + π_1_	μ + τ_F_ + π_2_ + γ_GF_

Here, μ is a general mean, the τ terms represent treatment effects, the π terms represent period effects, and the γ terms represent the treatment × period interaction.

When F is no treatment, τ_F_ and γ_FG_ are null and the model of the group I is as follows:

**Table:** 

	Period 1	Period 2
Group I (FG)	μ + π_1_	μ + τ_G_ + π_2_

Suppose the ratio of the period 2 to the period 1 is 1:*n*. The constancy of $\frac{{{\text{d}}S}}{{{\text{d}}t}}$ means that the period effect π_1_ is constant during the period 1. Under the condition of state steadiness which is confirmed by the (*n* + 1) times observations during the period 1, when the period 2 follows successively the period 1 and *n* is sufficiently large, we can postulate that the π_2_ is almost equal to (π_1_ + π_1_/*n*) in the group I. The larger *n* is, the more we can believe the steadiness of the state and the approximation of π_2_. Then the difference of the response between the two periods is (τ_G_ + π_1_/*n*) and we can measure τ_G_ with the repeated observations of group I. Thus steady state trials are considered as variants of cross-over trials.

### Prerequisite for steady state trials

How many figures should we adopt for *n*? In the above model, we postulate that $\frac{{{\text{d}}Z}}{{{\text{d}}t}}$ is equal to *k*, or π_2_ is equal to (π_1_ + π_1_/*n*). When *n* is infinitely large, this postulation is reasonable. However, when *n* is moderately large, the postulation receives criticism. There are many biological parameters which show cyclical or periodic variations, for example follicle-stimulating hormone or luteinizing hormone levels in female blood plasma. Another criticism is that the observed variable might reach the critical point after the steady state and change drastically without exposures. Before executing steady state trials in medicine, we have to examine biologically the trial condition for the possibility of cyclical or drastic state change. If some period ratio is thought to be critical, we have to avoid using such *n* for steady state trials.

## Discussion

We have deduced steady state trials (SSTs) from the counterfactual model, from which randomized controlled trials (RCTs) were also deduced. Although RCTs are thought to be paramount trials in recent clinical research, STTs can also offer the valid method to measure causal effects, when the state before the exposure is steady and large changes are immediately observed. The smaller the ratio of the after-period to the before-period is, the better we can rely on the measurement of the causal effect. When the after-period is relatively long, the measurements of SSTs may be confounded and RCTs should be considered in such situations. RCTs are also necessary when outcomes long after the exposure are important, even if SSTs show causal effects immediately.

Individual causal effects are defined as a contrast of the counterfactual outcomes. Because only one of those values is observed, it has been proposed that individual causal effects cannot be identified in epidemiological research [18, 19]. The epidemiologic principle is that a person may be exposed to an agent and then develop disease without there being any causal connection between exposure and disease [9]. SSTs show that we can measure individual causal effects in the condition where repeated observations are performed, the state before the exposure is steady, and large changes are immediately obtained after the exposure. This approach could open the door to the individual causal inference and other conditions for the individual causation that should be researched in epidemiology.

One example of steady state trials is parachute use in skydiving [5]. At the height of 4000 m, we jump into the sky and we are falling at the terminal velocity of 55 m/s after a few seconds. Within 3 s after opening parachutes, we usually fall at the next terminal velocity of 5 m/s. Now we record acceleration values at the interval of 3 s. Once we have the terminal velocity of 55 m/s, the acceleration value of 0 m/s^2^ is observed about twenty times before opening parachutes and the deceleration value of 17 m/s^2^ for 3 s is observed one time after opening parachutes. The *Y*_imp_ is the deceleration value of 17 m/s^2^ for 3 s. After sampling one successful skydiving, the 95 % credible interval of the posterior distribution Δ with prior Beta(0.5, 0.5) is computed as 0.12–0.99. In 2010, 1308 members of the United States Parachute Association (USPA) reported skydiving injuries requiring medical attention [20]. During the same year, USPA members and first-time students made roughly 3 million jumps. These data may be translated into the following sample distribution.

$$ \begin{gathered} {\text{Under the exposure }}\quad 2 9 9 8 6 9 2 { }\,Y_{\text{imps}} {\text{ out of 3}}000000{\text{ trials}} \hfill \\ {\text{Under the non-exposure}}\quad {\text{No }}Y_{\text{imp}} {\text{ out of 3}}000000 \times 20{\text{ trials}} \hfill \\ \end{gathered} $$

We adopt Beta(0.5, 0.5) as the prior distributions for *θe* and *θu*. However, the huge sample size fixes practically the same posterior distribution as when we believe the prior probability of *θu* is almost null. The 95 % credible interval of Δ is computed as 0.9995–0.9996 with WinBUGS. Classical statistics show that the 95 % confidence interval of the safe skydiving proportion is 0.99954–0.99959.

SSTs are practicable in the situation where immediate clinical responses are important, such as in the emergency room, where confounders are under the control of practitioners. Many treatments in emergency medicine have a long good history of SSTs in innumerable persons and can be regarded as scientific medical interventions without RCTs, such as intravenous injection of glucose for patients in hypoglycemic coma, injection of adrenalin (epinephrine) for patients with anaphylactic shock, a tourniquet for bleeding patients, and so on.

For example, one-person SST is presented in the use of adrenalin injection for an imaginary adult patient with anaphylactic shock. The data of the patient is shown in the Table 1. The systolic blood pressures (SBP) were recorded at the interval of 1 min. The patient had an intravenous injection of 0.1 mg adrenalin at time 10 min. We first check whether $\frac{{{\text{d}}S}}{{{\text{d}}t}}$ in the period *B* follows normal distribution. If there are any outliers, SSTs is not the choice for this trial. The data of the table can be considered as following normal distribution. The mean of (*k* + δ_*is*_) is one. The estimated variance of (*k* + δ_*is*_) is

**Table 1:** Systolic blood pressure in an imaginary adult patient with anaphylactic shock

Time (min)	*S* (mmHg)	d*S*/d*t* (mmHg/min)
0	50	
1	50	0
2	49	−1
3	50	1
4	53	3
5	53	0
6	54	1
7	56	2
8	57	1
9	58	1
10	60	2
Exposure		
11	92	32

Systolic blood pressure of the patient was recovering slowly without treatments. The patient had an intravenous injection of 0.1 mg adrenalin at time 10 min

*S* Systolic blood pressure

[(−1)^2^ + (−2)^2^ + 0^2^ + 2^2^ + (−1)^2^ + 0^2^ + 1^2^ + 0^2^ + 0^2^ + 1^2^]/(10 − 1) = 1.33.

The standard error of [(*Y* − *X*)/*A* − (*k* + δ_*is*_)] is √[1.33(1/1 + 1/10)] = 1.21.

Two-sample *t* statistic is (32 − 1)/1.21 = 25.6,

which follows the *t* distribution on (1 + 10 − 2) = 9 degrees of freedom. The *P* value is computed as <10^−8^. In this statistical estimation, we postulate that the distribution of (*Y *– *X*)/*A* follows the normal distribution with the same variance as σ^2^ which is estimated by the sample variance of (*k* + δ_*is*_). However, we do not really have data for the estimation of the variance of (*Y *– *X*)/*A*. We propose the above *P* value as an informal index to be used as a measure of discrepancy between (*Y* – *X*)/*A* and (*k* + δ_*is*_). We recommend that this *P* value should be smaller than 0.001 to show discrepancy. SBP over 90 mmHg is a clinically important value in shock, and we can consider that $\frac{{{\text{d}}S}}{{{\text{d}}t}}$ of 32 mmHg/min for 1 min after the exposure is *Y*_imp_. With prior Beta(0.5, 0.5) for *θe* and *θu*, the 95 % credible interval of the posterior distribution Δ is computed as 0.09–0.99. When we have the prior information that *Y*_imp_ have not been observed in the past unexposed states for 1000 min in total, we can adopt Beta(0.5, 1000.5) as the prior distribution for *θu* and *n* can be smaller to infer causal effects, if we confirm that unexposed state in the period *B* is in the same condition as the previously reported unexposed states. After observing one success of *Y*_imp_ after the exposure, with prior Beta(0.5, 0.5) for *θe*, prior Beta(0.5, 1000.5) for *θu* and *n* = 1, the 95 % credible interval of the posterior distribution Δ is computed as 0.15–1.00. Practically, *n* will be more than two in order to confirm the equal conditions, even when we adopt almost null prior for *θu*. When we execute SSTs in population studies, if some period ratio is thought to be critical, the participants are divided into two or three groups to which different values of *n* are allocated. RCTs between the groups are also possible in this setting.

SSTs are also possible in treatment trials of neurodegenerative diseases whose patients almost always show progressive deteriorations. For example, although RCTs has not been performed in the levodopa therapy of Parkinson’s disease [21], neurologists will admit that symptoms lasting for several months of early Parkinson’s patients almost always improve within a few days after receiving levodopa.

In preventive medicine, oral rehydration therapy is effective against diarrhea [22]. RCTs have compared oral rehydration with intravenous hydration [23]. SSTs can offer the measurement of the effect difference between the treated and the non-treated in acute stage diarrhea. Because of the necessity of controlling confounders, SSTs may be restricted within a narrow set of research topics in preventive medicine. However, if we have sufficient past databases of disease incidence and the derivative of the incidence rate with respect to time is constant, we may use SSTs for the measurement of effects of exposures which have a latent period of several years.

## Appendix

The essence of Bayes theorem is as follows. Suppose *θ* is some quantity that is currently unknown, and let *p*(*θ*) denote the prior distribution of *θ*. Suppose we have some observed evidence *y*, whose probability of occurrence is assumed to depend on *θ*. This dependence is formalized by *p*(*y*|*θ*), known as the likelihood. The posterior distribution taking account of the evidence *y* is denoted *p*(*θ|y*). Bayes theorem is

$$ p(\theta |y) \propto p(y|\theta ) \, \times p(\theta ). $$

The beta distribution is a conjugate family for the binominal likelihood. This means that a beta prior distribution and a binominal sample distribution provide a beta posterior distribution by Bayes theorem.

**Table:**

Prior distribution	Beta(α, β)
Observed sample proportion	*r* successes out of *n* trials
Posterior distribution	Beta(α + *r*, β + *n* – *r*)

The mean of Beta(α, β) is $\frac{{{\upalpha}}}{{{{\upalpha}} + {{\upbeta}}}}$.
